# Lignocellulosic Membranes Grafted with *N*-Vinylcaprolactam Using Radiation Chemistry: Load and Release Capacity of Vancomycin

**DOI:** 10.3390/polym16040551

**Published:** 2024-02-18

**Authors:** Maite Rentería-Urquiza, Guadalupe Gabriel Flores-Rojas, Belén Gómez-Lázaro, Felipe López-Saucedo, Ricardo Vera-Graziano, Eduardo Mendizabal, Emilio Bucio

**Affiliations:** 1 Departamento de Química, Centro Universitario de Ciencias Exactas e Ingenierías, Universidad de Guadalajara, Blvd. M. García Barragán #1451, Guadalajara 44430, Mexico; maite.renteria@academicos.udg.mx (M.R.-U.); eduardo.mmijares@academicos.udg.mx (E.M.); 2 Departamento de Química de Radiaciones y Radioquímica, Instituto de Ciencias Nucleares, Universidad Nacional Autónoma de México, Circuito Exterior, Ciudad Universitaria, Mexico City 04510, Mexico; belenlazaro98@gmail.com (B.G.-L.); felipelopezsaucedo@gmail.com (F.L.-S.); 3 Instituto de Investigaciones en Materiales, Universidad Nacional Autónoma de México, Mexico City 04510, Mexico; graziano@unam.mx; 4 Facultad de Ciencias, Campus El Cerrillo Piedras Blancas, Universidad Autónoma del Estado de México, Carretera Toluca-Ixtlahuaca Km 15.5, Toluca 50200, Mexico

**Keywords:** lignocellulose, bio-membrane, skin maguey, biopolymer, grafting, thermal responsiveness, gamma rays, membranes, *N*-vynilcaprolactam, *Agave salmiana*, drug release, cuticles

## Abstract

Radiation chemistry presents a unique avenue for developing innovative polymeric materials with desirable properties, eliminating the need for chemical initiators, which can be potentially detrimental, especially in sensitive sectors like medicine. In this investigation, we employed a radiation-induced graft polymerization process with N-vinylcaprolactam (NVCL) to modify lignocellulosic membranes derived from *Agave salmiana*, commonly known as maguey. The membranes underwent thorough characterization employing diverse techniques, including contact angle measurement, degree of swelling, scanning electron microscopy (SEM), atomic force microscopy (AFM), Fourier-transform infrared-attenuated total reflectance spectroscopy (FTIR-ATR), nuclear magnetic resonance (CP-MAS ^13^C-NMR), X-ray photoelectron spectroscopy (XPS), and uniaxial tensile mechanical tests. The membranes’ ability to load and release an antimicrobial glycopeptide drug was assessed, revealing significant enhancements in both drug loading and sustained release. The grafting of PNVCL contributed to prolonged sustained release by decreasing the drug release rate at temperatures above the LCST. The release profiles were analyzed using the Higuchi, Peppas–Sahlin, and Korsmeyer–Peppas models, suggesting a Fickian transport mechanism as indicated by the Korsmeyer–Peppas model.

## 1. Introduction

In recent years, there has been a growing interest and significant progress in the research and development of materials based on natural and semi-synthetic polymers. These materials offer promising properties, including biocompatibility [1,2], biodegradability [3,4], hydrophilicity [5,6], excellent mechanical properties [7,8], and low density [9]. These inherent characteristics make these materials highly suitable for a broad spectrum of applications across various sectors. Notably, they have proven beneficial in fields such as medicine [10], food packaging [11,12], microelectronics [13,14], and other cutting-edge technologies like fuel cells [15,16,17]. In addition to the excellent inherent properties of natural polymer materials, they also possess the flexibility to be tailored and modified to provide added value, customizing them for specific applications. These modifications can introduce stimulus-responsive properties, rendering them suitable for diverse applications such as sensors [18] or controlled and prolonged release systems for antimicrobial agents [19].

Controlled or prolonged drug release has significant potential in various medical applications, particularly in topical treatments [20,21]. Consequently, innovative methodologies in materials engineering are emerging to develop more efficient and biodegradable materials with promising prospects. The design of these novel materials is highly versatile and can be achieved through modification using graft polymerization techniques [22]. This approach introduces new chemical or stimulus-responsive properties to the base material [23], which can be very advantageous in drug delivery systems based on polymeric matrices, enhancing their biological efficacy [24,25].

In this context, poly(*N*-vinylcaprolactam) (PNVCL) has demonstrated its efficacy in modifying various matrices through graft polymerization techniques [26,27]. PNVCL imparts a thermal-responsive characteristic to the matrix due to its temperature-sensitive nature, undergoing a transition from hydrophilic to hydrophobic behavior in response to the temperature of the aqueous solution. This phenomenon, known as the lower critical solution temperature (LCST), has found practical applications across various domains. In medical applications, especially those involving drug loading and release, PNVCL’s LCST aligns conveniently with skin temperature (33 °C) [28,29,30].

Graft polymerization can be accomplished through various techniques, but gamma radiation-induced graft polymerization stands out as highly effective in initiating free radicals in polymer matrices with low reactivity [31,32]. These free radicals initiate a chain polymerization reaction, offering control over graft percentage by adjusting monomer concentration and absorbed dose [33]. An advantage of this method is its independence from pre-functionalization or the addition of other chemicals that could potentially harm the matrix before graft polymerization [34]. Therefore, gamma radiation-induced graft polymerization has the capability to produce graft polymers without necessitating additives or chemical initiators that might alter the final material’s properties and, particularly in medical applications, pose toxicity risks leading to adverse side effects [35,36].

This research article presents the gamma radiation-induced graft polymerization of *N*-vinylcaprolactam (NVCL) onto lignocellulosic membranes of natural origin obtained from the skin of *Agave salmiana*, commonly known as maguey. Subsequently, studies were conducted to evaluate the capacity of these new materials for loading and releasing vancomycin, a widely used antimicrobial agent. The new materials underwent comprehensive characterization through various spectroscopic techniques, including FTIR-ATR, CP-MAS ^13^C-NMR, and XPS, as well as through SEM and AFM techniques. Additionally, the thermal response of the modified membranes, water retention, and hydrophilicity were studied by measuring the contact angle with water droplets, and mechanical properties were determined through uniaxial tension tests.

## 2. Materials and Methods

Lignocellulosic membranes, sourced from the skin of *Agave salmiana* and denoted as MS, possess a thickness of 104 ± 5.4 μm and a density of 0.05 mg cm^−3^. These membranes underwent a series of treatments, beginning with immersion in a basic solution containing 0.1 M NaOH for 24 h at 40 °C. Subsequently, they were soaked in a mixture of water and methanol (1/1 v %) for an additional 24 h at 25 °C. This pretreatment aimed to eliminate any excess biomass and soluble residues, including arabinoses, cutin, saponins, hemicelluloses, glucans, etc., resulting in a membrane composed of insoluble compounds (cellulose, lignocellulose and cutan residues) [37]. Vancomycin was obtained from Fagron (Chía, Colombia), and sodium hydroxide, *N*-vinylcaprolactam (NVCL), and methanol were sourced from Sigma Aldrich (St. Louis, MO, USA). The monomer NVCL underwent distillation under reduced pressure before utilization.

### 2.1. Grafting of Membranes (MS-g-NVCL)

Segments of pretreated lignocellulosic membrane (MS), sized at 1.2 cm × 4 cm, were carefully positioned in an ampoule containing 9 mL of an *N*-vinyl caprolactam (NVCL) solution with varying concentrations of the monomer (ranging from 0–80 g/v %). This solution was meticulously prepared using a mixture of water and methanol (1/1 v %). To eliminate any traces of air, argon was bubbled through the solution for 20 min, followed by sealing of the ampoule. Subsequently, the sealed ampoules were exposed to a ^60^Co gamma-ray source at different absorbed doses, spanning from 0 to 60 kGy. Following gamma radiation exposure, any residual monomer and homopolymer in the membrane were removed by immersing the grafted membrane (MS-g-NVCL) in a water/methanol solution (70/30 v %) for 24 h. The membrane was then subjected to vacuum drying at 40 °C until a consistent weight was achieved. The grafting percentage (G %) was determined using Equation (1):

G (%) = 100 [(W_f_ − W_i_)/W_i_] (1)

where W_f_ and W_i_ are the weights after and before the graft, respectively.

### 2.2. Contact Angle

The inner and outer contact angles of the dry membranes (1.2 cm × 3 cm) were measured at different locations at predefined times of 1 and 5 min using the Kruss DSA 100 drop shape analyzer and its built-in software, with each experiment being repeated in triplicate.

### 2.3. Study of Swelling: Thermal-Response

The MS and MS-g-NVCL membranes were submerged in 20 mL of water at various temperatures, spanning from 20 to 55 °C, for a duration of 24 h. Afterward, the membranes were extracted, any surplus water was meticulously absorbed using filter paper, and their weights were recorded. The lower critical solution temperature (LCST) of the membranes was determined by evaluating the extent of swelling at each temperature, calculated using the following Equation (2):

Swelling (w %) = 100 [(W_w_ − W_d_)/W_d_] (2)

here, W_d_ represents the weight of the films before swelling, and W_w_ corresponds to the weight after swelling.

### 2.4. Mechanical Tests

The mechanical characteristics of the membranes were evaluated through uniaxial tensile tests. Wet membranes were molded into dumbbells and then vacuum-dried before the tests, following the procedure outlined in the ASTM D1708 standard. All experiments were conducted in triplicate using an INSTRON 1125 universal testing machine (Instron Inc., Norwood, MA, USA) with a crosshead speed of 10 mm/min.

### 2.5. Load and Release of Vancomycin

Membrane pieces (MS and MS-g-NVCL), each measuring 1 cm × 1.2 cm, were placed in vials containing 4 mL of a pH 7 vancomycin solution (50 µg/mL). Monitoring drug loading was carried out at specific time intervals by withdrawing samples from the medium and measuring the UV absorbance of the solution at 280 nm at room temperature. Following this, the aliquot was returned to the solution. The quantity of loaded vancomycin was calculated using Equation (3), obtained from a calibration curve and the surface area of the membrane utilized. The experiment was conducted in triplicate.

Loaded drug (µg/cm^2^) = (A_1_ − A_2_)/0.0159084 (3)

where A_1_ and A_2_ denote the initial and final absorbance values of the loading medium, respectively.

The assessment of drug release involved transferring vancomycin-loaded membranes into vials containing 4 mL of pH 7.4 phosphate buffer at both 25 °C and 40 °C. Monitoring of drug release was carried out through UV spectroscopy, with absorbance measurements at 280 nm taken at predetermined intervals. The drug concentration was then determined using a calibration curve. These experiments were carried out in triplicate.

Furthermore, the drug release kinetics from both the MS and MS-g-NVCL membranes were analyzed using various representative mathematical models [38,39,40,41], as described by the following Equations (4)–(6):

Higuchi release model: M_t_ = K_h_t^1/2^ (4)

here, M_t_ represents the fraction of drug released at each time (t), and K_h_ denotes the Higuchi kinetic constant. Additionally, the drug release mechanism was explored through the semi-empirical Korsmeyer–Peppas and Peppas–Sahlin models (Equations (5) and (6)).

(5) $$\mathrm{Korsmeyer}–\mathrm{Peppas}\;\mathrm{model}:\;M_{t}=K_{1}t^{n}$$

(6) $$\mathrm{Peppas}–\mathrm{Sahlin}:\;M_{t}=K_{1}t^{n}-{K_{2}t}^{2n}$$

here, K_1_ represents the contribution of Fickian diffusion that considers the structural and geometric characteristics of the device, K_2_ represents the contribution of relaxation of the polymer system, and n is the release exponent, offering insights into the release mechanism. A value of n at 0.45 or less corresponds to Fickian diffusion. If it falls between 0.45 and 0.89, it indicates non-Fickian transport. A value of n equal to 0.89 represents Case II transport, while a value greater than 0.89 signifies a super Case II transport mechanism [42].

### 2.6. Instruments

Vacuum Drying:

The Yamato ADP21 oven model facilitated vacuum drying and storage of samples for a minimum of 24 h at 25 °C and 0.1 kPa before utilization.

Contact Angle Measurement:

Contact angles on both sides (inner and outer) of the membrane were measured using the Kruss DSA 100 droplet shape analyzer (Matthews, NC, USA). The measurements were conducted on dry flat samples (1.2 cm × 3 cm) at various locations, all at a temperature of 25 °C.

FTIR-ATR Analysis:

Dry samples of MS and MS-g-NVCL membranes were analyzed on both sides (inner and outer) using a Perkin-Elmer Spectrum 100 spectrometer (Norwalk, CN, USA) with 16 scans. The TA Universal analysis 2000 software was employed for data processing.

Nuclear Magnetic Resonance Spectroscopy (CP-MAS ^13^C-NMR):

The CP-MAS ^13^C-NMR spectra of the membranes were recorded on a 300 MHz Bruker Avance II spectrometer (75.48 MHz for ^13^C). The analysis involved spin rates of 5 kHz and 4 kHz, using 4 mm zirconia rotors. Tetramethylsilane (TMS) served as an external reference, and Mnova 14 software facilitated spectral data analysis.

Scanning Electron Microscopy (SEM):

Images were obtained using the Zeiss Evo LS15 instrument (Jena, Germany) on both sides (inner and outer) of the membrane. Small samples of 1 cm in length were cut, coated with gold, and analyzed under high vacuum.

Atomic Force Microscopy (AFM):

Images were acquired using the JEOL Scanning Probe Microscope model JSPM-4210 instrument on both sides (inner and outer) of the membrane. Small samples of 0.5 cm in length were cut and tested in contact mode.

X-Ray Photoelectron Spectroscopy (XPS):

Sample spectra were recorded on both sides (inner and outer) of the membranes using a Microprobe PHI 5000 Versa Probe II (Chanhassen, MN, USA). An Al Kα monochromatic excitation source (energy 1486.6 eV, 100 µm beam diameter) and a Multi-Channel Detector (MCD) were utilized. The energy scale was corrected using the C1s peak to 285.0 eV, and Multipack 9.9 software was used for spectral data analysis.

Mechanical Tests:

An INSTRON 1125 (Instron Inc., Norwood, MA, USA) universal tensile testing machine was used with a crosshead speed of 10 mm/min.

Ultraviolet–Visible (UV-Vis):

UV spectra were obtained using an Agilent model 8453 Spectrophotometer (Waldbronn, Germany) with quartz cuvettes of 1 cm length.

## 3. Results and Discussion

### 3.1. Synthesis of MS-g-NVCL Membranes

**Figure 1:**
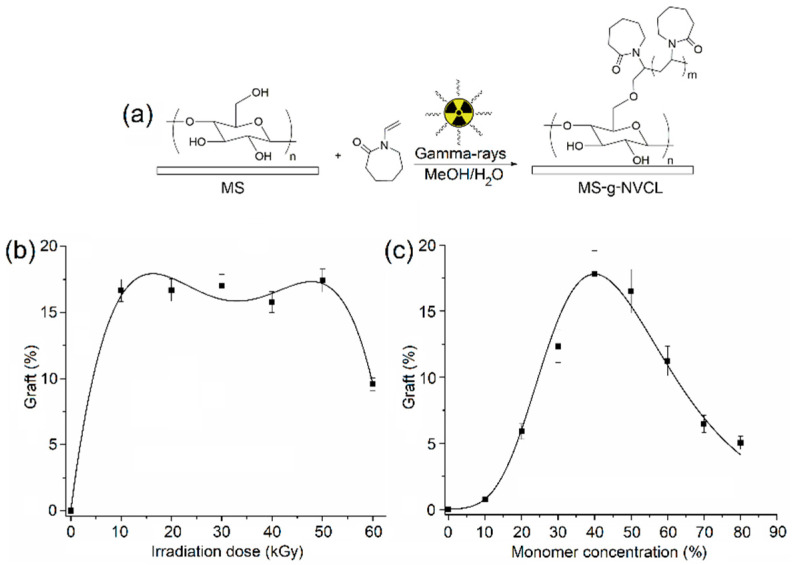
(**a**) Illustration of gamma ray-induced graft polymerization. (**b**) Grafting percentage versus irradiation dose with a monomer concentration of 50 g/v %. (**c**) Grafting percentage versus monomer concentration at an irradiation dose of 20 kGy.

The PNVCL grafting onto MS membranes was effectively conducted through gamma radiation to induce the graft polymerization reaction, explored across various absorbed doses and monomer concentrations (Figure 1a). Examination of the impact of the absorbed dose on the grafting percentage revealed a maximum grafting level of approximately 17%, achieved with irradiation doses ranging from 10 to 50 kGy and a monomer concentration of 50%. However, escalating irradiation doses led to a reduction in the grafting percentage due to material degradation, evident in the loss of material weight.

Conversely, when investigating the influence of monomer concentration at an absorbed dose of 20 kGy, it was observed that the grafting percentage peaked at a concentration of 40%. Beyond this concentration, a consistent decline in the grafting percentage occurred with increasing monomer concentration. This observation implies that concentrations exceeding 40% favored homopolymerization, resulting in a reduction in the grafting degree (Figure 1c). In summary, these studies indicate the feasibility of achieving the maximum grafting degree with a low irradiation dose, such as 20 kGy, and a monomer concentration of 40%, while mitigating undesired secondary reactions like homopolymerization and matrix degradation.

### 3.2. Water Contact Angle and Thermal Responsiveness

**Figure 2:**
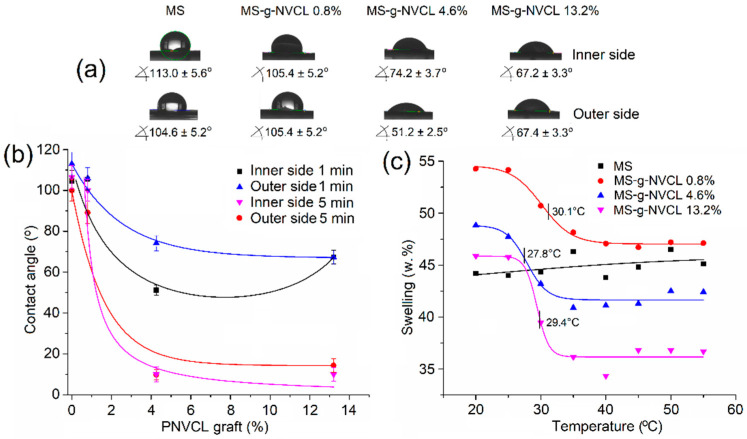
(**a**) Images of contact angle at 25 °C, 1 min; (**b**) relationship between the water contact angle at 25 °C and the PNVCL grafting percentage onto MS membranes, with the contact angles recorded in both sides of the membrane at 1 and 5 min; and (**c**) thermal responsiveness of MS-g-NVCL membranes with different grafting percentages. The LCST is indicated in the graph.

The hydrophilic properties and thermal responsiveness of MS-g-NVCL membranes were evaluated by measuring the contact angle and water swelling at various temperatures. Contact angles were determined on both sides of the membranes at 25 °C, revealing comparable hydrophilicity to pristine membranes (MS) (Figure 2a,b).

However, the initial average contact angle of 103.8° decreased with the degree of grafting, suggesting increased hydrophilicity. For instance, at a grafting degree of 13.2%, the average contact angle reduced to 67.3°, indicating an enhanced capacity of the material to absorb water at 25 °C, as corroborated by the thermal response studies.

On the other hand, the thermal response was evaluated in MS-g-NVCL membranes with different degrees of grafting: 0.8%, 4.6%, and 13.2%. They exhibited LCST values of 30.1 °C, 27.8 °C, and 29.4 °C, respectively, indicating that the grafting percentage can modify the thermal response of the material. In contrast, the MS membrane did not display any thermal response and only showed a slight increase in its degree of swelling (Figure 2c).

### 3.3. FTIR-ATR Study

The pristine MS membrane was subjected to infrared analysis, unveiling the presence of diverse chemical functional groups, such as –OH (1461 and 3316 cm^−1^), –NH– (3372 cm^−1^), –C–H (2995 cm^−1^), –C=O– (1733 and 1642 cm^−1^), and –COC– (1161 cm^−1^). Interestingly, variations were noted between each side of the MS membrane, with fewer hydrophilic functional groups (–C=O–, –OH, and –NH–) detected on the outer side, aligning with the results of contact angle measurements and subsequent studies.

**Figure 3:**
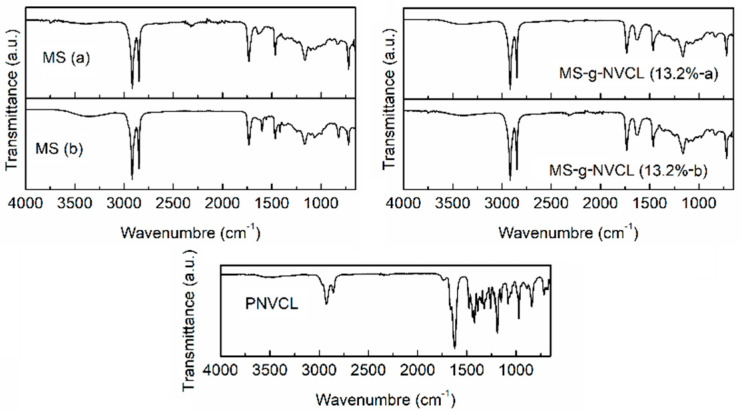
FTIR-ATR spectra of both sides of the MS and MS-g-NVCL (13.2 %) membranes, and PNVCL obtained by gamma rays. Samples labeled with (a) and (b) are the inner and outer side, respectively.

In the MS-g-NVCL spectra, a distinctive band associated with the functional group –C=O– (1633 cm^−1^) was evident, along with bands in the range of 2856 to 2927 cm^−1^ and 1233 cm^−1^ related to –C–H and –C–N–, these prominent bands provided unequivocal evidence of the successful grafting of PNVCL onto MS. This was further corroborated by the FTIR spectra of PNVCL, which displayed the characteristic band at 1636 cm^−1^, attributed to the –C=ON– group (Figure 3).

### 3.4. CP-MAS ^13^C-NMR and XPS Analysis

**Figure 4:**
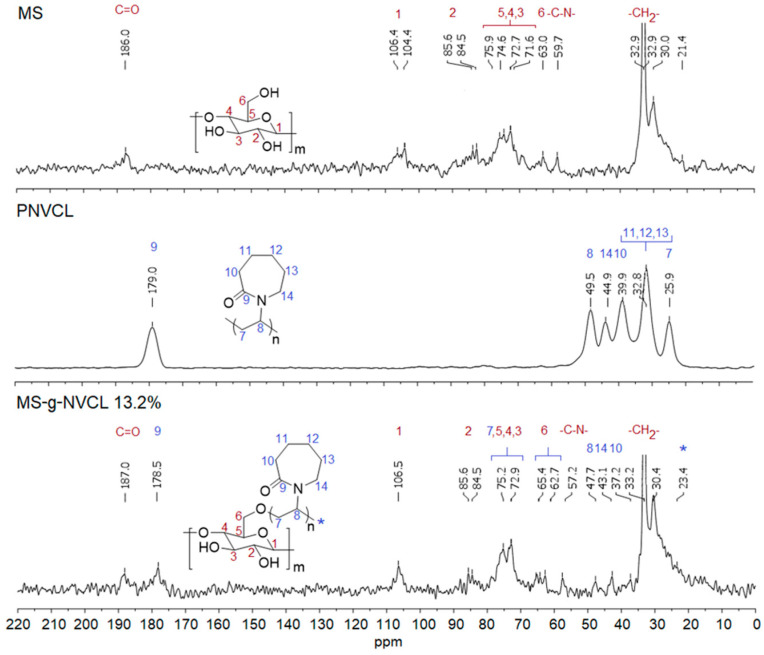
CP-MAS ^13^C-NMR spectra of MS, PNVCL homopolymer, and MS-g-NVCL (13.2%) membranes. C-*, polymer aliphatic chain carbon.

The CP-MAS ^13^C-NMR spectra of the MS membrane revealed signals from cellulose chains and the presence of aliphatic chains (–CH_2_–CH_2_–) at 21.4–35.0 ppm, linked to hydroxyl and amino groups (–CH_2_–OH, –N–CH_2_–) at 59.7 ppm and carbonyl groups (–C=O–) at 186.0 ppm, from non-soluble compounds (e.g., lignin and cutan) [43,44]. These results were supported by other analyses such as infrared and XPS studies. The signal at 63.0 ppm corresponds to –CH_2_OH, while signals ranging from 71.6 to 106.4 ppm were attributed to the endocyclic carbons of cellulose [19,33,45], as illustrated in Figure 4.

The spectrum of the MS-g-NVCL membranes confirms the modification through graft polymerization, displaying characteristic signals of PNVCL. Among these signals, the most distinctive corresponds to the carbonyl group assigned to the amide group of the *N*-vinyl caprolactam units, located at 178.5 ppm, and that of the carbons associated with the ring and aliphatic chain of the polymerized vinyl units at 37.2–47.7 ppm (Figure 4).

**Figure 5:**
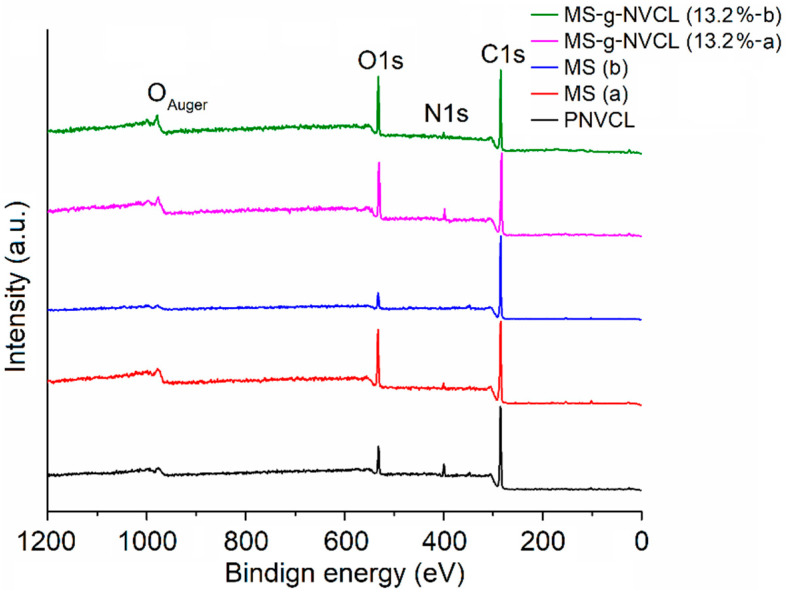
XPS survey spectra of membranes of both inner (labeled with a) and outer (labeled with b) sides with different grafting percentages and PNVCL.

The XPS analysis provided insights into the atomic compositions of both sides of MS and MS-g-NVCL membranes, showcasing distinct peaks for carbon (C1s at 285.0 eV), oxygen (O1s at 531.0 eV), and nitrogen (N1s at 399.4 eV), as illustrated in Figure 5. Calculating the areas under the C1s, O1s, and N1s peaks in the XPS survey spectra, using appropriate sensitivity factors, determined the atomic percentage of the membranes. The proportion of carbon and oxygen for each side of the membrane is detailed in Table 1. The outcomes revealed an increase in the O1s and N1s proportions on both sides of the MS-g-NVCL membranes, aligning with the grafting of PNVCL and the development of a coating on the membrane by the grafted PNVCL chains. Additionally, the results indicated a consistent atomic percentage on both sides of the grafted membrane, signifying a uniform graft polymerization across the matrix’s surface.

**Figure 6:**
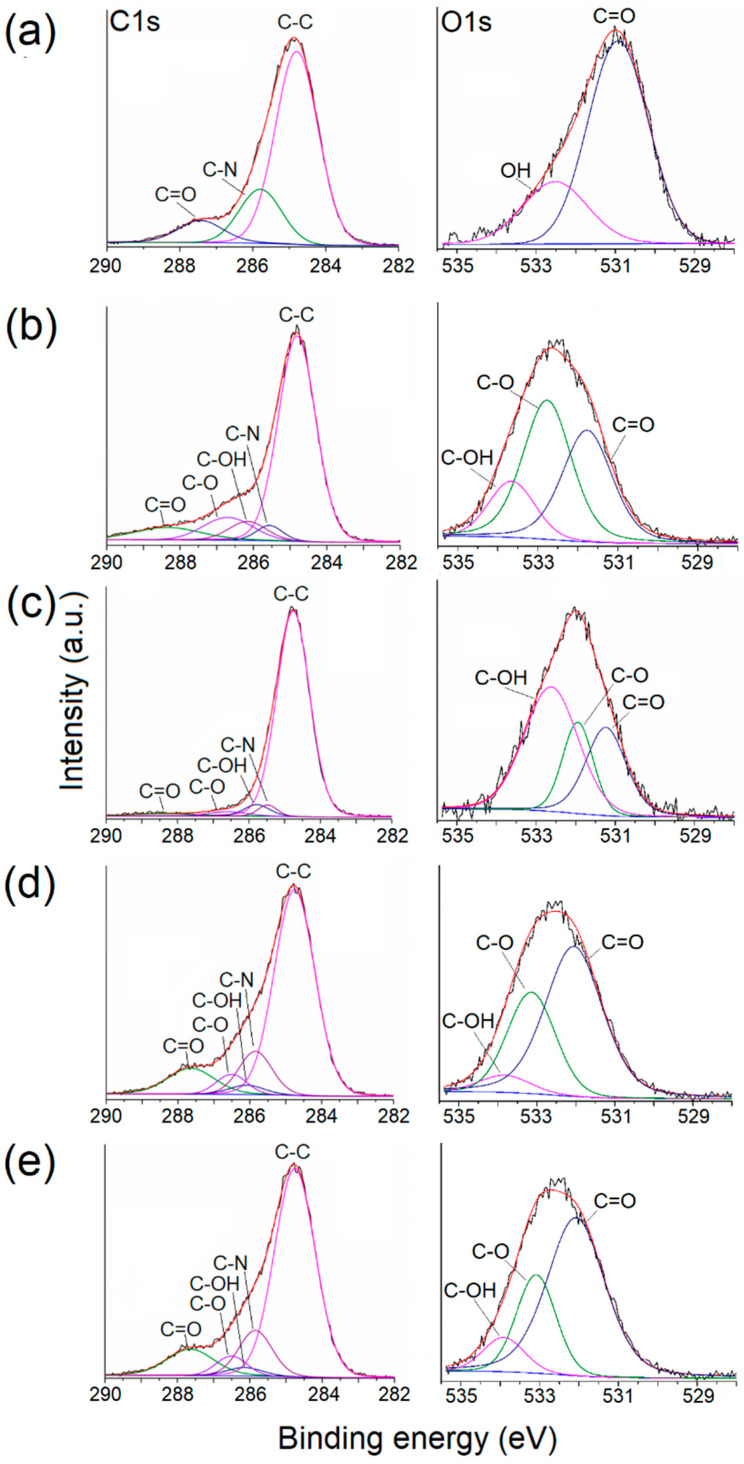
Deconvolution of XPS spectra corresponding to C1s and O1s of (**a**) PNVCL and (**b**) and (**c**) the inner and outer side of the MS membrane; (**d**) and (**e**) correspond to the inner and outer side of the MS-g-NVCL (13.2%) membrane, respectively.

The C1s spectrum of PNVCL exhibited three distinct peaks: C=O, C–N, and C–C. Simultaneously, the O1s spectrum featured two peaks: C=O and –OH, corresponding to the oxygen of the carbonyl group and the oxygen from water molecules enclosed in the polymer. In contrast, the spectra of MS and MS-g-NVCL membranes were resolved into five peaks: C=O, C–O–C, C–OH, C–N, and C–C. Notably, there was an increase in the C–N peak in the MS-g-NVCL membranes, rising from an average of 8.6% to 15.1%. Furthermore, the O1s spectra displayed three deconvolution peaks assigned to C–O–C, –OH, and C=O. These findings imply a reduction in the peak associated with –OH, indicating that grafting primarily occurred on the –CH_2_–OH segment derived from cellulose, along with backbone cleavage of cellulose [19,33]. Lastly, the N1s spectrum revealed a central binding energy level at 399.83 eV (Figure 6 and Table 1).

### 3.5. Mechanical Properties

**Figure 7:**
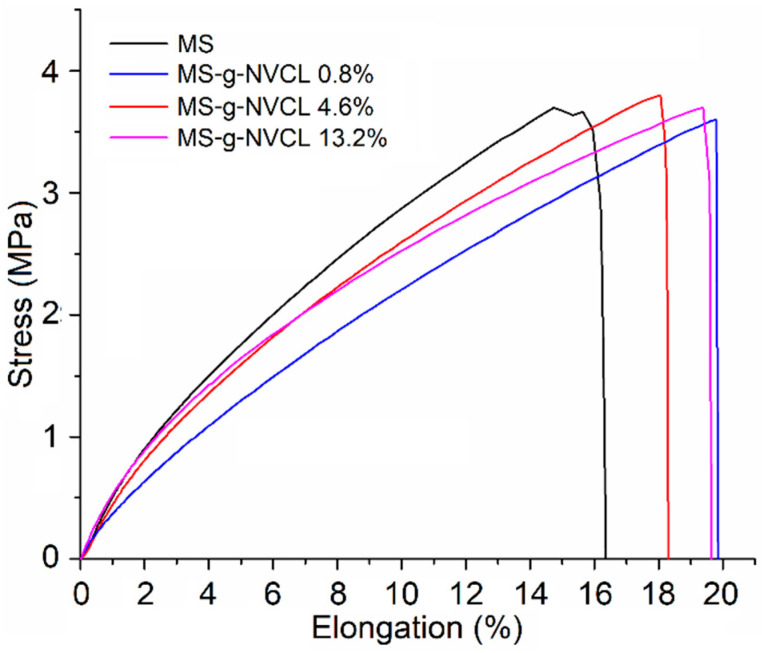
Tensile stress–strain curves of MS and MS-g-NVCL membranes with different graft percentages.

The mechanical properties of the membranes with different graft percentages ranging from 0 to 13.2% were evaluated (Figure 7). The results revealed that there were no significant changes in these properties with varying graft percentages. The elongation at the break point remained within a range of 16.8% to 20.2%. This behavior was similarly observed for the Young’s modulus and tensile break, with the MS-g-NVCL membrane having a grafting percentage of 0.8% showing the lowest Young’s modulus and tensile break. However, as the grafting percentage increased, these values became more similar to those of the pristine membrane (Table 2). These results contrast with previous reports where *Agave salmiana* lignocellulose membranes grafted with 4-vinylpyridine, which exhibited a slight increase in Young’s modulus and tensile strength and marginal decrease in elongation at break [19].

### 3.6. SEM and AFM Study

**Figure 8:**
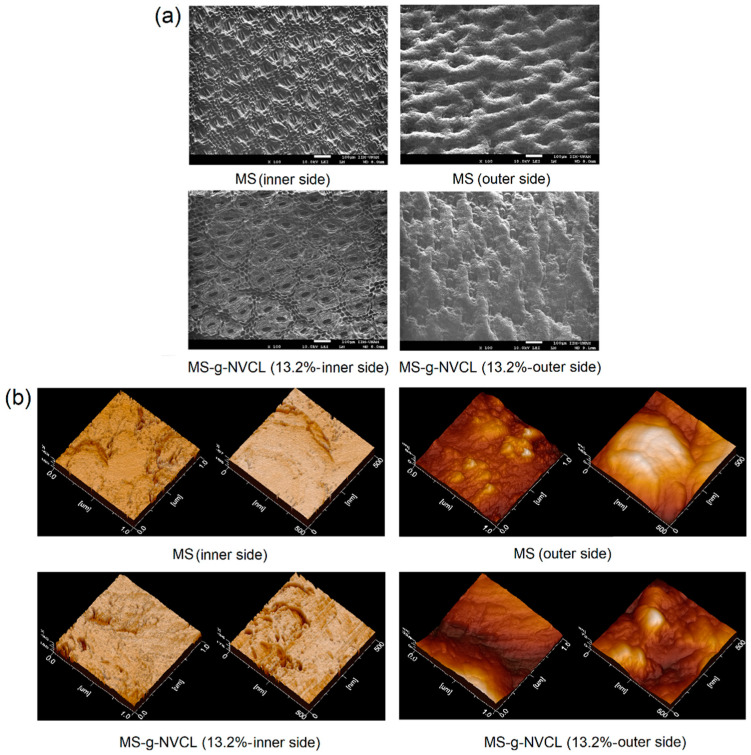
(**a**) SEM and (**b**) AFM images of MS and MS-g-NVCL (13.2%) membranes with different magnifications of the inner and outer sides.

SEM and AFM images unveiled an intricate surface morphology of MS membranes (Figure 8a). The membrane’s inner side (labeled with a) displayed a structure characterized by stomata (30–55 µm long and 11–25 µm wide, and density of 57.6 stomata mm^−2^) with a tetracytic structure presenting a suprastomatal and substomatal chamber [46,47], integral to the plant’s water and nutrient absorption system, as well as waste removal, gas exchange, and barrier against pathogens. Conversely, the outer side (labeled with b) exhibited fibers (50–75 µm) with a mesh-like pattern with small channels. Following grafting polymerization, the SEM images of the membranes (MS-g-NVCL) showed no substantial modifications at a micrometer level. Likewise, AFM images on a nanometer scale did not indicate appreciable changes on the membrane’s surface. These results imply that the modification through PNVCL grafting was uniform on both sides of the membranes, and there was no apparent degradation or damage caused by gamma radiation or grafting polymerization under the applied conditions (monomer concentration at 30% and 20 kGy). These findings align with the observed mechanical properties.

### 3.7. Loading and Release of Vancomycin

**Figure 9:**
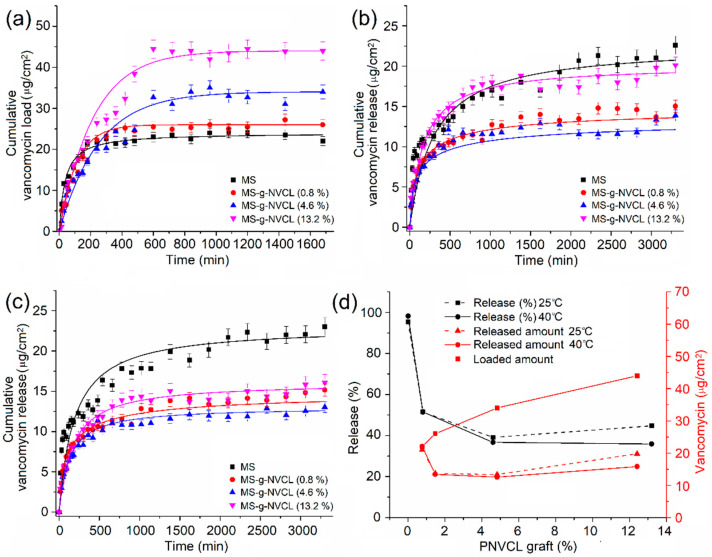
Kinetics of (**a**) loading of vancomycin at 25 °C, (**b**) release of vancomycin at 25 °C, (**c**) release of vancomycin at 40 °C, and (**d**) percentage and amount of drug released versus graft percentage of PNVCL at 3060 min.

Vancomycin loading was carried out using MS and MS-g-NVCL membranes with variable graft percentages at 25 °C and a pH of 7.0, while the release was studied at 25 °C and 40 °C with a pH of 7.4. The drug loading process in the MS-g-NVCL reached the maximum loading at approximately 600 min, in contrast to the MS membrane, which reached this point at approximately 400 min (Figure 9a). These findings demonstrate the effect of PNVCL polymeric chains on loading time, which resulted in slower drug loading, possibly due to steric hindrance. However, PNVCL grafting provided a significant increase in vancomycin loading, increasing from 22 µg cm^−2^ for 0% graft membranes to 44 µg cm^−2^ for those with 13.2% graft (Figure 9d). Figure 9b,c show that the vancomycin release rate at 25° and 40 °C for all membranes was initially rapid, then gradually decreased; after approximately 1000 min, the release rate was slow.

**Figure 10:**
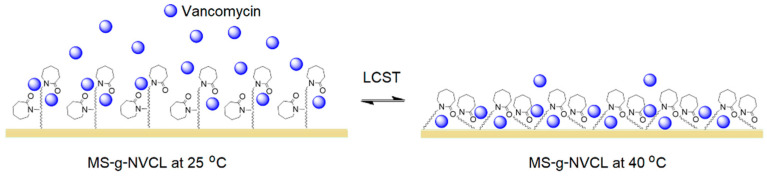
Illustration depicting the influence of the LCST of PNVCL graft on the release of vancomycin.

The presence of the graft decreased the release rate, which is desirable as it allows for longer drug dosing time, and that rate can be modified with the degree of grafting. (Figure 9b,c). Figure 9d shows that at 25 °C, at 3060 min, the MS membrane released 95.4% of the drug, the membrane with a grafting percentage of 4.6% released 39.1%, and the membrane with a grafting percentage of 13.2% increased the amount released to 44.7%, possibly due to saturation of the membrane surface with the vancomycin. These results indicate that the PNVCL graft provides a sufficient number of amide groups capable of interacting with the –C=O, NH, and –OH groups of the drug, causing a decrease in the release rate. When the temperature was increased from 25 to 40 °C, the release rates remained practically the same, except for the membrane with the highest amount of graft (13.2%), where a decrease in the release rate was observed, obtaining a release rate close to that of those with lower grafting. This decrease is attributed to the collapse of the PNVLC chains because at 40 °C they are above their LCST, causing the vancomycin molecules to be trapped inside the graft chains due to their transition from hydrophilic to hydrophobic, making their release into the medium more difficult (Figure 10). These results indicate an increase in surface drug encapsulation from 3.7% (4.6% grafting) to 8.8% (13.2% grafting) at 40 °C (Figure 9d).

**Figure 11:**
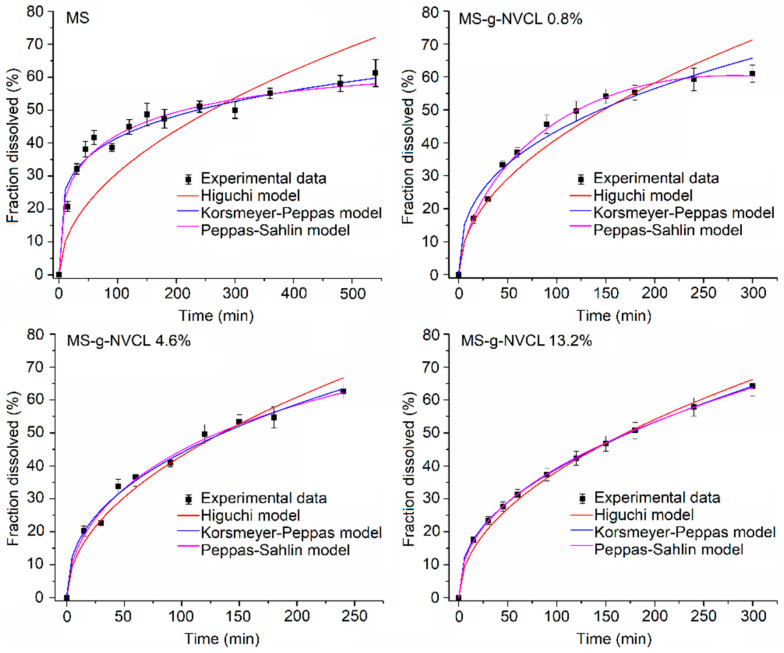
Fitting of mathematical models of drug release at 60% for MS and MS-g-NVCL membranes at 25 °C with grafting percentages of 0.8%, 4.6%, and 13.2%.

Figure 11 illustrates the adequate fit of the Higuchi, Peppas–Sahlin, and Korsmeyer–Peppas models to the grafted membranes. However, the Peppas–Sahlin and Korsmeyer–Peppas models have a better fit to the experimental data and present higher R^2^ values than the Higuchi model (Table 3). In all instances, the Korsmeyer–Peppas model proposes a Fickian drug release mechanism, substantiated by the “n” values (Fickian transport ≤ 0.45) [42]. This mechanism is attributed to the release of vancomycin through the stomata of the membranes. Consequently, the Fickian mechanism, coupled with the thermal response, results in controlled and extended vancomycin release. Although the Peppas–Sahlin model fits slightly better the release profiles, the small and negative K_2_ values indicate that the polymer relaxation mechanism contributes virtually nothing to the drug release. On the other hand, in MS-g-NVCL (0.8%) and MS-g-NVCL (13.2%) membranes, the “n” values derived from the Peppas–Sahlin model indicate a release system characterized by a flat geometry, in this case a membrane, exhibiting non-Fickian transport [48,49], these results contrast with those obtained by the Korsmeyer-Peppas model. Higuchi’s model does not fit the experimental data of the ungrafted membrane (MS), showing R^2^ values below 0.6, implying that the drug release from the ungrafted membrane follows a different mechanism compared to those of the grafted membranes.

## 4. Conclusions

The successful synthesis of MS-g-NVCL materials involved the grafting of PNVCL onto lignocellulosic membranes using gamma radiation, achieved with low monomer concentrations and absorbed doses. This grafting process conferred thermal responsiveness to the membrane, with an LCST range of 27.8–30.1 °C. Uniform PNVCL grafting on both sides of the membrane was evidenced by a similar contact angle. Microscopy and spectroscopy studies confirmed the modification of the lignocellulosic membrane through PNVCL grafting facilitated by gamma radiation. FTIR-ATR and CP-MAS ^13^C-NMR analyses highlighted characteristic signals in MS and MS-g-NVCL membranes, such as the carbonyl amide group and increased nitrogen atoms from the grafted PNVCL chains according to XPS.

Regarding mechanical properties, no significant changes were observed in MS membranes after PNVCL grafting. However, the PNVCL modification influenced both the loading amount and the rate of vancomycin release from the membranes, suggesting a Fickian drug release mechanism as indicated by the Korsmeyer–Peppas model. Furthermore, PNVCL grafting enabled prolonged sustained release, potentially reaching therapeutic levels, by reducing the drug release rate at temperatures above its LCST. This paves the way for designing advanced therapeutic materials for treating chronic or postoperative wounds.

## Figures and Tables

**Table 1:** XPS elemental composition and chemical group concentration results for PNVCL homopolymer, MS, and MS-g-NVCL membranes on both inner (a) and outer (b) side.

	Elemental Composition (%) *			Chemical Group Concentration (%) *							
				C1s					O1s		
Membrane	C1s	N1s	O1s	C=O	C–O–C	C–OH	C–N	C–C	C=O	–OH	C–O–C
PNVCL	81.1	6.9	11.9	14.7	0	0	22.6	62.6	56.3	43.6	0
MS (a)	75.5	3.4	20.7	12.4	13.8	11.6	9.9	52.0	34.6	28.5	36.8
MS (b)	90.1	0.8	9.9	6.9	6.9	8.3	7.4	70.3	32.6	35.6	31.7
MS-g-NVCL (13.2%-a)	73.6	4.3	22.1	14.0	10.0	9.1	15.0	51.8	41.2	25.5	33.1
MS-g-NVCL (13.2%-b)	69.9	3.5	26.4	13.9	10.3	8.8	15.3	51.4	42.0	26.6	31.2

* ASF content in Multipack code estimated uncertain at about 5%.

**Table 2:** Mechanical properties of MS and MS-g-NVCL membranes under uniaxial tension.

Membrane	Young’s Module (MPa)	Tensile Break (MPa)	Elongation Break (%)
MS	48 ± 2.26	3.7 ± 0.24	16.8 ± 1.16
MS-g-NVCL 0.8%	36 ± 3.74	3.6 ± 0.58	20 ± 3.78
MS-g-NVCL 4.6%	45 ± 3.82	3.8 ± 0.87	18.5 ± 4.96
MS-g-NVCL 13.2%	48 ± 4.74	3.7 ± 0.68	20.2 ± 2.55

**Table 3:** Results of the mathematical models (Higuchi, Peppas–Sahlin, and Korsmeyer–Peppas) for the vancomycin release profiles of MS and MS-g-NVCL with different graft percentages at 25 °C and 40 °C. The values were obtained for up to 60% release of vancomycin.

Membrane	Higuchi				Peppas–Sahlin								Korsmeyer–Peppas					
	25 °C		40 °C		25 °C				40 °C				25 °C			40 °C		
	R^2^	K_h_^a^	R^2^	K_h_^a^	R^2^	K_1_	K_2_	n ^b^	R^2^	K_1_	K_2_	n ^b^	R^2^	K_1_	n ^b^	R^2^	K_1_	n ^b^
MS	0.54	3.1	0.55	3.2	0.96	12.04	-0.59	0.33	0.97	11.88	−0.57	0.32	0.96	15.52	0.21	0.97	15.35	0.21
MS-g-NVCL 0.8%	0.93	4.1	0.91	4.2	0.99	3.28	-0.04	0.63	0.99	3.46	−0.04	0.62	0.97	7.74	0.37	0.98	7.71	0.36
MS-g-NVCL 4.6%	0.97	4.3	0.98	4.3	0.99	4.91	-0.07	0.51	0.99	5.18	−0.07	0.50	0.99	6.43	0.42	0.98	6.31	0.42
MS-g-NVCL 13.2%	0.97	3.8	0.97	3.8	0.98	5.65	0.64	0.33	0.98	5.05	1.25	0.30	0.98	5.19	0.44	0.98	5.16	0.43

^a^ Higuchi-release kinetic constant. ^b^ Exponent of Fickian diffusion. K_1_ represents the contribution of Fickian diffusion and K_2_ represents the contribution of relaxation of the polymer system.

## Data Availability

The data presented in this study are available on request from the corresponding author.
